# Adolescent Enrollment in Psychosocial Care: Do Parents Make a Difference?

**DOI:** 10.3390/ijerph17197066

**Published:** 2020-09-27

**Authors:** Katerina Paclikova, Zuzana Dankulincova Veselska, Andrea Madarasova Geckova, Jitse P. van Dijk, Sijmen A. Reijneveld

**Affiliations:** 1 Olomouc University Social Health Institute, Palacky University in Olomouc, 711 11 Olomouc, Czech Republic; katerina.paclikova@oushi.upol.cz (K.P.); andrea.geckova@upjs.sk (A.M.G.); j.p.van.dijk@umcg.nl (J.P.v.D.); 2 Graduate School Kosice Institute for Society and Health, Pavel Jozef Safarik University in Kosice, 040 11 Kosice, Slovakia; 3 Department of Health Psychology and Research Methodology, Faculty of Medicine, Pavel Jozef Safarik University in Kosice, 040 11 Kosice, Slovakia; 4 Department of Community and Occupational Medicine, University Medical Center Groningen, University of Groningen, 9713 AV Groningen, The Netherlands; s.a.reijneveld@umcg.nl

**Keywords:** adolescence, emotional and behavioral problems, psychosocial care, parental characteristics

## Abstract

Care for adolescents with emotional and behavioral problems (EBP) is frequently unequally distributed. Parents may play a role in the access to this care. Therefore, the aim was to explore the association between parental characteristics and their adolescent’s enrollment in psychosocial care. We used data from the Care4Youth cohort study. Our sample consisted of 446 adolescents (mean age 13.22 years, 48% boys) and 382 parents (mean age 42.95 years, 14% males). EBP combined with enrollment created four groups: 1, no EBP/no care; 2, no EBP/care; 3, EBP/no care; 4, EBP/care. We assessed differences in parental characteristics among the groups. Group 2 had a significantly lower socioeconomic position (*p* < 0.01), more psychological distress (*p* < 0.001), poorer supervision (*p* < 0.001) and lower family social support (*p* < 0.05) than Group 1. Group 4 had a significantly lower socioeconomic position (*p* < 0.01) and poorer supervision (*p* < 0.001) than Group 1. Group 3 had significantly poorer supervision (*p* < 0.001) than Group 4. The poor supervision in Group 3 requires attention, as these adolescents receive no care. The quality of parental supervision should be addressed generally, e.g., by providing better parenting support and more parental training.

## 1. Introduction

Emotional and behavioral problems (EBP) have a high prevalence in adolescence (in Europe, approximately 10–20% of adolescents are suffering from EBP), and if left untreated, may have negative long term consequences for the adolescents, their families and society as a whole [1,2]. These problems can lead to poor educational outcomes, failure to finish compulsory education, long-term unemployment, poor interpersonal relationships and parenting difficulties in adulthood [2,3,4]. In addition, these problems may be associated with severe alcohol and drug abuse, engagement in risky sexual behavior, self-harm and involvement in the social justice system [4,5,6,7,8]. Development of more severe emotional and behavioral disorders in adulthood, such as anxiety, depression and obsessive-compulsive disorder, might start with EBP in adolescence and be due to a wide range of factors ranging from genetic predispositions and trauma to family characteristics and parental skills [4,9,10,11,12].

Care for adolescents with EBP is often unequally distributed, with some adolescents not receiving any care. The percentage of adolescents with EBP who never received care for their EBP varies across studies from about 30% [13,14] up to more than a half [15]. Parental perceptions of their adolescent’s need for care may play a role in adolescent enrollment in care. Jansen et al. [16] found a difference between adolescents having EBP and parents perceiving their adolescent’s need for receiving care for these problems. Of the adolescents in this study whose parents reported a need for care, 71% never received care. There was also a small number of adolescents (2.4%) whose parents did not report a need for care, but who still received care. However, evidence is lacking on the comparison of adolescents inside and outside care, and the problems they have.

Parents typically have an important role in seeking care for their adolescent, and whether the problems of adolescents lead to the use of care for these problems also seems to depend at least partially on the characteristics of the parents, such as their socioeconomic position, psychological distress, parenting skills and perceived social support. In various studies, the low socioeconomic position of the family was associated with adolescents’ use of school-based care [17], social care [18] and general and psychosocial care [19]. Parents perceive that their adolescents with EBP need care more frequently when they themselves report having psychological problems, having used care for these problems before [16,20] or experiencing stress [14]. The enrollment of adolescents into psychosocial care is also associated with poor parenting skills [21] and low family social support [21,22].

Previous research has examined which parental characteristics are associated with the enrollment of adolescents into psychosocial care for their EBP and with not getting appropriate care when they have EBP (treatment gap). However, evidence is lacking on the factors associated with other combinations of enrollment and having EBP—adolescents without EBP but enrolled in psychosocial care as well as those without EBP and not enrolled in psychosocial care. Our study adds a more thorough insight into the factors associated with all possible combinations of EBP and care in adolescents.

Therefore, the aim of the present study was to explore the association between parental characteristics and their adolescent’s enrollment or non-enrollment in psychosocial care. Adolescents with or without EBP were included. The combination of two characteristics was used (enrollment vs. non-enrollment in psychosocial care and having vs. not having EBP) to create four different groups among which parental characteristics were compared.

## 2. Materials and Methods

### 2.1. Sample and Procedure

We used data from the baseline measurement wave of the Slovak Care4Youth cohort study. Participants were parents and adolescents from the community sample as well as from psychosocial care. The respondents had to meet the following criteria: children should be aged 10–16 years, children and their parents/legal representatives should be able to understand the Slovak language and should be able to fill out the questionnaires on their own, and they should come from the Kosice region in Eastern Slovakia.

**Figure 1:**
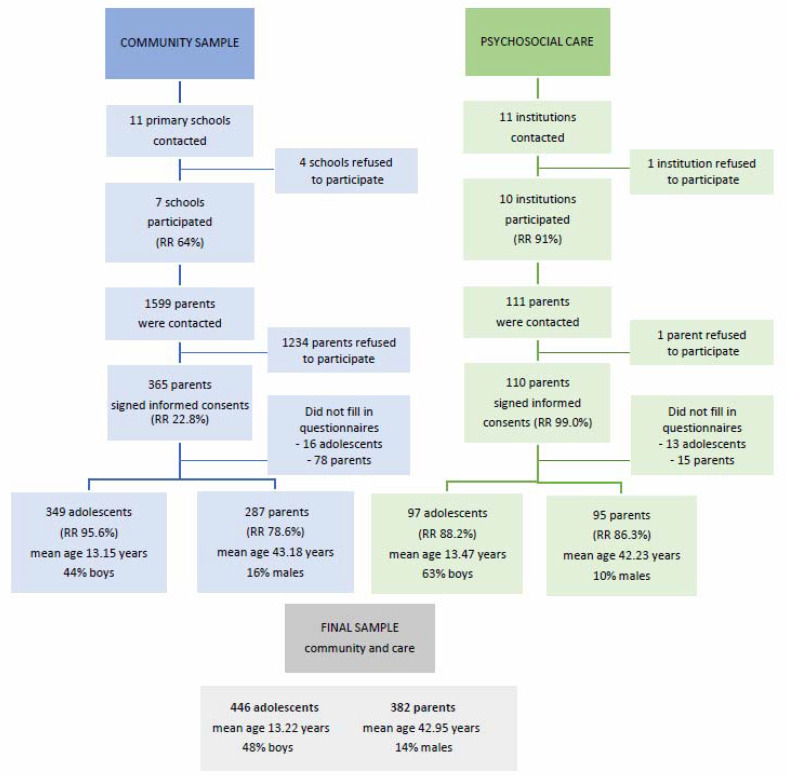
Flowchart outlining the process of recruitment of participants from the community and from psychosocial care services for the study, with basic descriptive information of the sample (RR—response rate).

Participants from the community (parents and their children) were recruited via randomly chosen primary schools in the Kosice region in Eastern Slovakia; they were approached from January until June 2017 via two-stage sampling. In the first stage, we contacted schools; in the second stage, the parents or legal representatives of the pupils were contacted. After being thoroughly informed of the consequences of participation in the study, parents were asked to provide us with signed informed consent on behalf of their children and themselves. Detailed information on response rates and the number of participants with basic descriptive information can be seen in Figure 1.

Participants from the care system (parents and their children) were recruited via institutions providing psychosocial care for adolescents with emotional and behavioral problems in the Kosice region in Eastern Slovakia that were approached from January 2017 until December 2018 via two-stage sampling. In the first stage, we contacted institutions; in the second stage, the parents or legal representatives of pupils were contacted. After being thoroughly informed of the consequences of participation in the study, parents were asked to provide us with signed informed consent on behalf of their children and themselves. Detailed information on the response rates and the number of participants with basic descriptive information can be seen in Figure 1.

For the purpose of this study, we used a combined sample consisting of 446 adolescents (mean age 13.22 years, 48% boys) and 382 parents (mean age 42.95 years, 14% males). The study was approved by the Ethics Committee of the Medical Faculty at Pavel Jozef Safarik University in Kosice (protocol 2N/2015).

### 2.2. Measures

A combination of two characteristics was used (having vs. not having EBP and enrollment vs. non-enrollment in psychosocial care) to create four different groups for which parental characteristics were compared. EBP were measured using the 20 difficulty items of the Strengths and Difficulties Questionnaire (SDQ), as reported by adolescents. The SDQ has 25 items [23], 20 of which are difficulty items. The response options were: 0, not true; 1, somewhat true; 2, certainly true. The summed scores of these 20 items range from 0 to 40, with a higher score indicating more problems. Cronbach’s α of the SDQ in our sample was 0.80. We dichotomized the SDQ scores into two categories: adolescents having problems (“abnormal”, score 20–40) and adolescents having no problems (“normal” and “borderline”; score 0–19) based on the recommended cut-off points [24]. Enrollment was regarded as being either in the care sample or being in the community sample and using care according to information provided by the parents. The combination of enrollment and EBP provided four possible categories: 1, no EBP and no care provided; 2, no EBP but care provided; 3, EBP but no care provided; 4, EBP and care provided.

Parent-reported socioeconomic position was measured with the MacArthur Scale of Subjective Social Status [25], which captures the common sense of position across the socioeconomic indicators (income, education and occupation). It presents a “social ladder” and asks individuals to mark the rung (0 to 10) on which they feel they stand, with a higher score meaning a better perceived socioeconomic position. We treated this variable as continuous.

Parent-reported psychological distress was measured with the General Health Questionnaire [26]. We used the 12-item version, which is scored on a 4-point Likert scale ranging from “better than normal”, through “same as usual” and “worse than usual” to “much worse than usual” for negatively worded items. Positively worded items were scored reversely. A higher score indicates a more severe condition. Cronbach’s α in our sample was 0.90.

Parent-reported perceived parenting style was measured on the Alabama Parenting Questionnaire—short version [27], which consists of nine items divided into three subscales: positive parenting, poor supervision and inconsistent discipline. Each subscale consisted of three items that were assessed on a 5-point Likert scale from “never” to “always”. A higher score in all subscales indicates the measured aspect of parenting style as being more prevalent. Cronbach’s α was 0.80 for the positive-parenting subscale, 0.68 for the poor supervision subscale and 0.61 for the inconsistent discipline subscale.

Parent-reported perceived social support was measured with the Multidimensional Scale of Perceived Social Support [28], which is a 12-item scale designed to measure perceived social support from three sources: family, friends and significant others. All items were assessed on a 7-point Likert scale ranging from “very strongly disagree” to “very strongly agree”. A higher score in each subscale (family, friends and significant others) means higher social support. Cronbach’s α was 0.95 for the family social support subscale, 0.95 for the friends social support subscale and 0.91 for the significant others subscale.

### 2.3. Statistical Analyses

As a first step, we described the background characteristics of the sample within the four groups based on enrollment in psychosocial care using descriptive statistics. Second, we explored the differences in parental characteristics among the four groups of enrollment in psychosocial care/EBP through one-way analyses of variance and Bonferroni post hoc tests. Statistical analyses were performed with SPSS v.20. We used power analyses in G-power to justify group sizes to ensure they had enough power to detect among-group differences [29].

## 3. Results

### 3.1. Background Characteristics of the Sample

The background descriptive characteristics of the sample and of the four groups of enrollment in psychosocial care/EBP are presented in Table 1. The percentage distribution of the groups of enrollment in psychosocial care/EBP was as follows: 59.7% no EBP and no care provided, 27.4% no EBP but care provided, 6.0 % EBP but no care provided and 6.9% EBP and care provided. Our sample and enrollment groups did not significantly differ regarding age or gender.

### 3.2. Comparisons among Groups

The enrollment in psychosocial care/EBP groups differed significantly regarding several parental characteristics (i.e., socioeconomic position, psychological distress, poor supervision and perceived family social support). Group 2 and Group 4 differed from Group 1, and Group 3 differed from Group 4. Group 2 had a lower socioeconomic position, more psychological distress, a higher score for poor supervision and lower perceived family social support than Group 1. Group 4 also had a lower socioeconomic position and a higher score for poor supervision than Group 1. Group 3 had a lower score for poor supervision (i.e., better supervision) than Group 4; see Table 2. We performed power analysis in G-power [29]. This showed that the sample sizes were sufficient to detect among-group differences with sufficient power (0.95).

## 4. Discussion

We explored the association of parental characteristics (i.e., socioeconomic position, psychological distress, parenting style and perceived social support), with adolescent enrollment or non-enrollment in psychosocial care when they have or do not have EBP. We found significant differences in parental characteristics regarding four groups of adolescent enrollment in psychosocial care with or without EBP. To our knowledge, this study is the first to compare four groups regarding adolescent enrollment in psychosocial care—those who are enrolled or not enrolled in psychosocial care with and without EBP.

Parents of adolescents who reported EBP and were enrolled in psychosocial care (Group 4) had a significantly lower socioeconomic position and poorer supervision than those without EBP and not in care (Group 1). Our findings are in line with previous studies showing low socioeconomic position and poor supervision to be associated with psychosocial problems in adolescents and a higher likelihood of enrollment in psychosocial care [17,18,19,21,30]. An explanation for these findings may regard the family stress model, which explains how economic disadvantage leads to worse parenting and psychological distress of parents, and this subsequently leads to worse developmental outcomes in adolescents [31]. Poorly supervised adolescents, probably due to less correction of their antisocial behavior, connect more with antisocial peers, have more psychosocial problems and enroll in psychosocial care more frequently [21,32]. Still, due to the cross-sectional design of our study, causality could not be clearly stated. Enrollment of an adolescent with EBP in psychosocial care may be the result of poor supervision but is not necessarily always so. On the contrary, a more demanding situation for parents might lead to worsening of their parental skills, resulting in poorer supervision. The low socioeconomic position of the family and poor supervision are both linked to a higher likelihood of psychosocial problems in adolescents and their consequent enrollment in care [33,34]. However, the direction of the causality remains to be determined in future longitudinal research.

Surprisingly, 27.4% of the adolescents in our sample did not report having EBP but were enrolled in psychosocial care (Group 2). Their parents had a significantly lower socioeconomic position, more psychological distress, poorer supervision and lower perceived family social support than those whose adolescents did not report having EBP and were not in psychosocial care (Group 1). Previous studies show that the abovementioned parental characteristics are associated with EBP in adolescents and the likelihood of enrollment in care [21,35,36]. Parental need for their adolescent’s enrollment in psychosocial care might come out of problems in the family and might not only be based on the problems of the adolescents [37]. Some parents might be overprotective or anxious about ensuring the safety of an adolescent. Other parents may exaggerate adolescent behavior and consider it problematic or transfer the responsibility for solving their adolescent’s problems to a care system. Another explanation may be the higher psychological distress of parents in this group especially. The mental health problems of parents might be reflected in their children, either genetically or by contextual influence. Thus their children may need psychosocial care, though not directly resulting in their EBP being detectable by an administrated questionnaire because of other protective factors [38]. Our finding of a rather high number of adolescents enrolled in psychosocial care without having EBP provides a new insight into the underlying process of using psychosocial care.

In our sample, 6% of adolescents were not enrolled in care, even though they reported having EBP. The unmet need for care based on existing problems has been widely explored in recent research and is referred to as a “treatment gap” [15,17,39,40]. Parents of adolescents with EBP in our sample who were not enrolled in psychosocial care (Group 3) had significantly better supervision than parents of adolescents who had EBP and were enrolled in psychosocial care (Group 4). Previous research shows better supervision (and other positive parenting skills) to be connected with fewer EBP in adolescence [31,41,42]. The question remains as to why adolescents who report having EBP in combination with their parents having better supervision do not enroll in psychosocial care. An explanation may be that parents with better supervision might have better parenting skills in general and are able to figure out the EBP of their adolescent on their own [21,42]. However, evidence about the association of good parental supervision and lower adolescent enrollment in psychosocial care is lacking.

Both inappropriate identification of an adolescent’s need for care and the inability to access care are likely to be connected with the unmet need for care of an adolescent. However, regarding certain characteristics of parents in our study, the lack of enrollment in care is supposed to stem more probably from inappropriate identification of the problems.

An important strength is the inclusion of adolescents with and without EBP who were enrolled in psychosocial care as well as those who were not enrolled. Having or not having EBP was measured with a well-validated instrument. Another strength is that we relied not only on information reported by the adolescent but also by the parents regarding parental characteristics and enrollment in care.

Some limitations should also be mentioned. Having or not having EBP was measured on the SDQ as reported only by adolescents. This might lead to inappropriately captured problems, even though it is a validated instrument. Adding the parental point of view and/or other instruments for measuring EBP used by professionals would be valuable. Moreover, because of the cross-sectional design of the study, we cannot be decisive as to causality. Thus, the associations of low socioeconomic position, psychological distress, poor supervision, low family social support and adolescents’ enrollment in psychosocial care may also partially have different causal paths.

Professionals and policymakers should be aware that some adolescents do not receive psychosocial care when they have EBP, whereas some adolescents receive psychosocial care even though they do not evidently have EBP. Furthermore, they should take into consideration that several parental characteristics are associated with an adolescent’s enrollment in psychosocial care. However, further research is needed to determine whether influencing parental characteristics and family factors would lead to a more accurate enrollment of adolescents in psychosocial care. Enrollment of an adolescent with EBP in psychosocial care may be the result of worse parental skills but, as was already discussed, it is not necessarily always so. On the contrary, a more demanding situation for parents might lead to worsening of their parental skills. Longitudinal research could shed more light on the pathways between parental characteristics and skills and enrollment in psychosocial care. Moreover, further research focused on a professional’s point of view might provide additional comprehension of the association between parental characteristics and an adolescent’s EBP, as well as their enrollment in psychosocial care.

## 5. Conclusions

A considerable number of adolescents have EBP but are not enrolled in psychosocial care; similarly, some adolescents do not report having EBP but are enrolled in psychosocial care. Several parental characteristics (i.e., socioeconomic position, psychological distress, poor supervision and family social support) are associated with adolescent enrollment and deserve attention regarding the reasons why adolescents are enrolling in psychosocial care. Considering parental characteristics may lead to more appropriate and more effective enrollment of adolescents with EBP into psychosocial care.

## Figures and Tables

**Table 1:** Background characteristics of the sample by enrollment in psychosocial care and environmental and behavioral problems (EBP) (350 parents and adolescents from care sample and community sample, collected in 2017–2018).

Variables	Group 1 No EBP and No Care Provided	Group 2 No EBP but Care Provided	Group 3 EBP but No Care Provided	Group 4 EBP and Care Provided	*p*-Value
Total number %	*n* = 209 59.7%	*n* = 96 27.4%	*n* = 21 6.0%	*n* = 24 6.9%	
Age of adolescents (mean, SD)	13.47 (1.41)	13.06 (1.65)	13.16 (1.24)	13.30 (1.71)	ns
Age of parents (mean, SD)	43.36 (4.91)	43.15 (7.88)	41.21 (4.37)	41.59 (4.86)	ns
Gender of adolescents Boys (%)	41.8%	54.2%	38.1%	54.2%	ns
Gender of parents Males (%)	15.8%	13.4%	16.7%	21.7%	ns

SD—standard deviation.

**Table 2:** Differences among of enrollment/EBP groups regarding parental characteristics: results of one-way analysis of variance and Bonferroni post hoc tests (350 parents and adolescents from the care sample and the community sample, collected in 2017–2018).

	Group 1 Mean (SD) *n* = 209	Group 2 Mean (SD) *n* = 96	Group 3 Mean (SD) *n* = 21	Group 4 Mean (SD) *n* = 24	*F*-Value	*p*-Value	Bonferroni Test Differences among Groups
Socioeconomic position *	6.86 (1.59)	6.22 (1.88)	6.44 (1.15)	5.78 (1.83)	4.76	0.003	1–2, 1–4
Psychological distress *	1.31 (2.56)	2.79 (3.45)	1.56 (2.94)	2.26 (2.30)	5.31	0.001	1–2
Parenting							
Positive parenting *	12.60 (1.68)	12.84 (1.61)	12.00 (1.72)	12.55 (2.30)	1.26	0.288	-
Inconsistent discipline *	7.85 (2.12)	7.96 (1.94)	7.83 (2.01)	7.73 (2.23)	0.10	0.962	-
Poor supervision *	4.83 (1.87)	5.70 (2.33)	4.72 (1.36)	6.68 (2.55)	7.83	0.001	1–2, 1–4, 3–4
Perceived social support							
Social support from family *	24.37 (4.17)	22.86 (5.40)	22.86 (5.37)	25.08 (3.55)	3.23	0.023	1–2
Social support from friends *	23.43 (4.43)	22.52 (5.18)	22.19 (5.88)	22.75 (5.65)	1.06	0.366	-
Social support from others *	25.35 (3.46)	24.48 (3.91)	23.62 (5.09)	25.79 (2.48)	2.63	0.051	-

SD—standard deviation. Group 1: no EBP and no care provided. Group 2: no EBP but care provided. Group 3: EBP but no care provided. Group 4: EBP and care provided. * A higher score means better socioeconomic position, higher psychological distress, better positive parenting, more inconsistent discipline, poorer supervision and better perceived social support from family, friends and others.
